# Cardioprotection by combination of three compounds from ShengMai preparations in mice with myocardial ischemia/reperfusion injury through AMPK activation-mediated mitochondrial fission

**DOI:** 10.1038/srep37114

**Published:** 2016-11-21

**Authors:** Fang Li, Xiaoxue Fan, Yu Zhang, Lizhi Pang, Xiaonan Ma, Meijia Song, Junping Kou, Boyang Yu

**Affiliations:** 1 Jiangsu Key Laboratory of TCM Evaluation and Translational Research, Department of Complex Prescription of TCM, China Pharmaceutical University, 639 Longmian Road, Nanjing, 211198, P.R. China

## Abstract

GRS is a drug combination of three active components including ginsenoside Rb1, ruscogenin and schisandrin. It derived from the well-known TCM formula ShengMai preparations, a widely used traditional Chinese medicine for the treatment of cardiovascular diseases in clinic. The present study explores the cardioprotective effects of GRS on myocardial ischemia/reperfusion (MI/R) injury compared with ShengMai preparations and investigates the underlying mechanisms. GRS treatment significantly attenuated MI/R injury and exhibited similar efficacy as Shengmai preparations, as evidenced by decreased myocardium infarct size, ameliorated histological features, the decrease of LDH production and improved cardiac function, and also produced a significant decrease of apoptotic index. Mechanistically, GRS alleviated myocardial apoptosis by inhibiting the mitochondrial mediated apoptosis pathway as reflected by inhibition of caspase-3 activity, normalization of Bcl-2/Bax levels and improved mitochondrial function. Moreover, GRS prevented cardiomyocytes mitochondrial fission and upregulated AMPKα phosphorylation. Interestingly, AMPK activation prevented hypoxia and reoxygenation induced mitochondrial fission in cardiomyocytes and GRS actions were significantly attenuated by knockdown of AMPKα. Collectively, these data show that GRS is effective in mitigating MI/R injury by suppressing mitochondrial mediated apoptosis and modulating AMPK activation-mediated mitochondrial fission, thereby providing a rationale for future clinical applications and potential therapeutic strategy for MI/R injury.

Myocardial ischemia/reperfusion (MI/R), one of the leading causes of human death worldwide, is due to blood restoration after a critical period of coronary artery obstructions, which is closely associated with cardiac dysfunctions, in particular myocardial infarction, left ventricular remolding and heart failure^1^. It is widely known that MI/R injury is a pathological process that includes augmented myocardial apoptosis and increased infarct size^2^. Therapeutic strategies aim at preventing cardiomyocytes apoptosis are of great clinical and health value for the treatment of MI/R injury^3^.

As a sensor of cellular energy status, AMP-activated protein kinase (AMPK) plays important roles in cell survival and apoptosis^4^. AMPK is a serine-threonine kinase involving in the regulation of energy balance and myocardial signaling in the heart^5^, and meanwhile exerting essential physiological functions including regulation of inflammation, mediation of reactive oxygen species (ROS) and protection from apoptosis^6^. Plenty literatures have reported that AMPK is cardioprotective during MI/R via promoting glucose uptake and glucose transporter translocation, inhibiting apoptosis and decreasing myocardial infarction^7^,^8^,^9^. In our previous study, we found that the YiQiFuMai powder injection, a traditional Chinese medicine prescription re-developed based on the well-known TCM formula Sheng-maisan, could provide significant cardioprotection against MI/R injury, and potential mechanisms might to suppression of cardiomyocytes apoptosis at least in part through activating the AMPK signaling pathways^10^. Recently, regulation of the cytoplasmic dynamin-related protein 1 (Drp1) emerges as a potential molecular target by which AMPK activation inhibits mitochondrial dysfunction and alleviates endoplasmic reticulum stress-associated endothelial dysfunction^11^. In stressed cells, Drp1 is recruited to the mitochondrial membrane and accelerates mitochondrial fission, therefore, resulting in mitochondrial malfunction^12^. Previous studies have revealed that AMPK activation could suppress high glucose-induced mitochondrial fission in endothelial cells^13^, suggesting the potential role of AMPK in the protection of mitochondrial function. Therefore, we hypothesize that pharmacological activation of AMPK could protect mitochondrial morphology and function through the regulation of Drp1 activation, thereby inhibit cardiomyocytes apoptosis.

In recent years, positive evidence from clinical trials has promoted acceptance of TCM for the treatment of cardiovascular diseases^14^,^15^,^16^. Combination therapy has been advocated for over 2000 years. Treatment that contain multiple drugs directing towards multi-pathways and multi-targets would possess stronger therapeutic efficacies for complex multifactorial diseases^17^. ShengMai San is a well-known traditional Chinese formula and its remedy preparations have already been widely used to prevent and cure cardio-cerebral ischemic diseases^18^,^19^,^20^. Recently, we screened a new drug combination (GRS, the proportion as 6:0.75:6) comprising three compounds, including ginsenoside Rb1 (Ginsenosides of Red ginseng-G), ruscogenin (Saponins of Radix ophiopogonis-R), and schisandrin (Lignans of Fructus schisandrae-S), which showed a significant cardioprotective effects in experimental studies^21^. The proportion of the combination was optimized to achieve the highest efficacy, quality stabilization and clinical safety for research^22^. However, its possible mechanism in action against MI/R injury remain unclear, and the equivalence of cardioprotective effects comparing with Shengmai preparations (YiQiFuMai powder injection, YQFM) still need further confirmation.

In the current study, therefore, GRS was tested *in vivo* in a MI/R injured mouse model, and *in vitro* in H9c2 cardiomyocyte cell line and primary cultured cardiomyocytes subjected to hypoxia/reoxygenation (H/R), investigating the therapeutic effect of GRS compared with YQFM and furthermore exploring the possible underlying mechanisms of GRS against MI/R injury, especially focusing on the mitochondrial fission by regulation of Drp1 phosphorylation (Ser 616) in an AMPK dependent manner.

## Results

### GRS decreased myocardial injury and improved the cardiac function in MI/R mice

**Figure 1:**
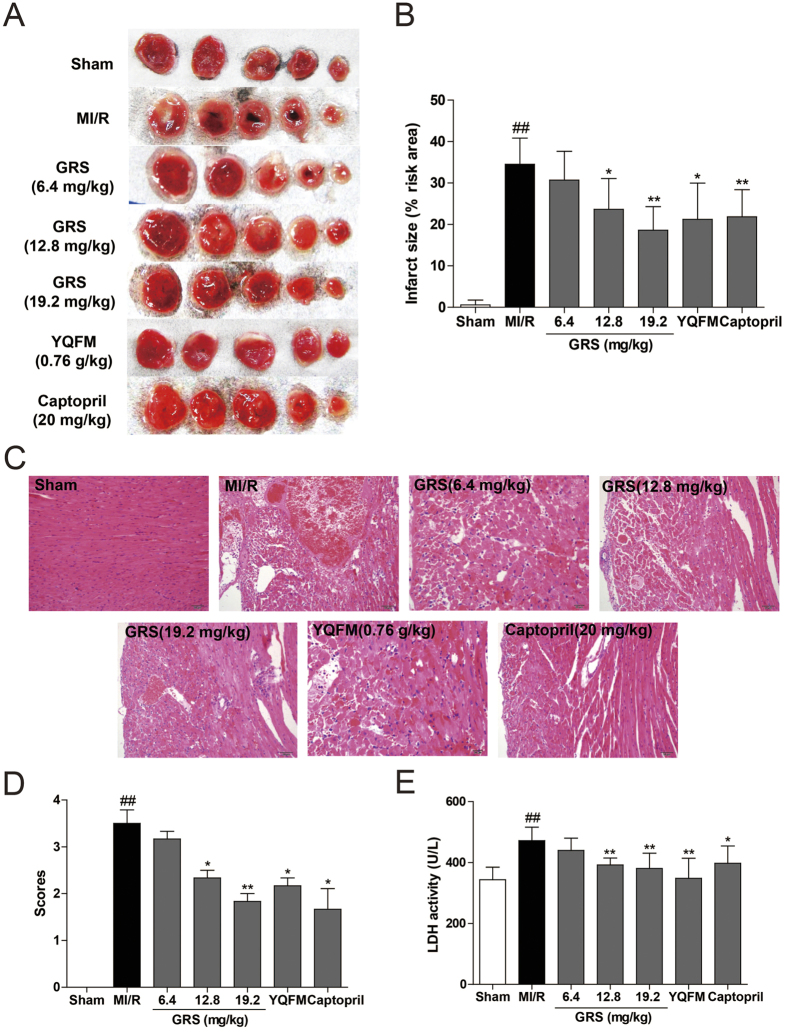
GRS decreased myocardial injury in MI/R mice. **(A)** 2,3,5-triphenyltetrazolium chloride (TTC) staining determined the effect of GRS on myocardial infarct area. **(B)** Graphic representation of myocardial infarct size. **(C)** Histopathological changes of representative myocardium sections were measured by HE staining (200×magnification). **(D)** Scores measurement of histopathological changes. **(E)** The release of LDH in serum at the end of reperfusion was determined. Results were presented as mean ± SEM. ^##^*P* < 0.01 vs. Sham group without MI/R, *P < 0.05, **P < 0.01 vs. group treated with MI/R alone. n = 6.

To investigated the effects of GRS on MI/R-induced injury, infarct size, histopathological examination and the release of LDH in serum were measured. As illustrated in Fig. 1, 30 min of ischemia and 24 h of reperfusion resulted in myocardial injury, as evidenced by increased infarct size, LDH activities and morphological alterations. However, GRS at the concentration of 6.4–19.2 mg/kg and captopril (20 mg/kg) treatment groups significantly suppressed the increase of infarct size (Fig. 1A,B). Simultaneously, the histopathological examination from MI/R mice’ heart tissues exhibited widespread myocardial structural disarray, increased necrosis and fusion area, and a large number of inflammatory cells infiltrating the myocardial tissue. By contrast, the histological features became typical of normal cardiac structure or mild architectural damage with distinct amelioration after GRS and captopril treatment (Fig. 1C,D). Following MI/R, GRS and captopril significantly reduced the LDH activity (Fig. 1E). Particularly, GRS at the highest concentration of 19.2 mg/kg exerted the equivalent myocardial protective effects compared to YQFM.

**Figure 2:**
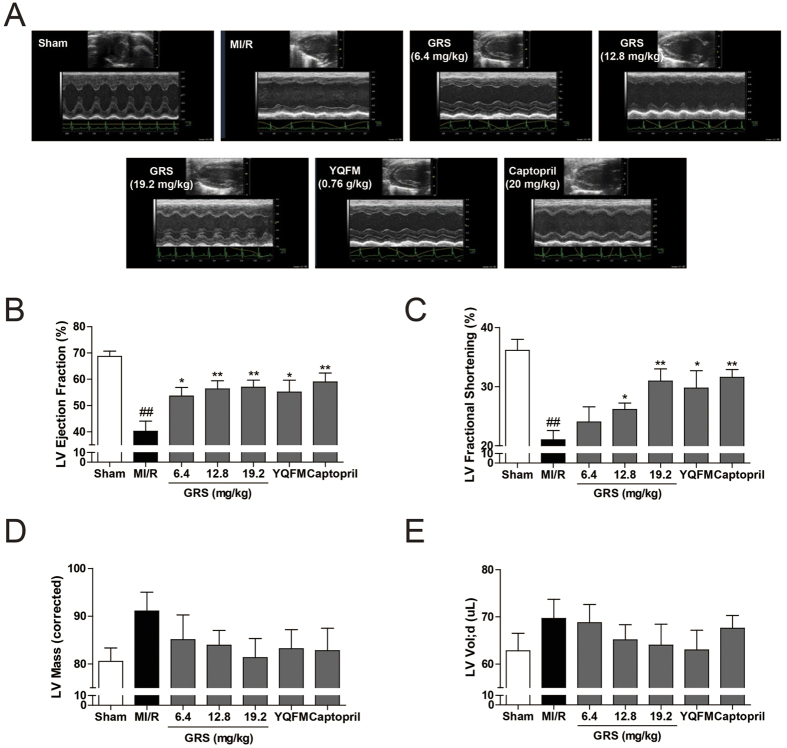
GRS improved the cardiac function in MI/R mice. **(A)** Representative images of echocardiography. Cardiac performance was determined by echocardiography 24 h after reperfusion. **(B–E)** LVEF values (**B**) LVFS (**C**) LV Mass (**D**) LV volumes (**E**) were measured by echocardiography. Results were presented as mean ± SEM. ^##^*P* < 0.01 vs. Sham group without MI/R, *P < 0.05, **P < 0.01 vs. group treated with MI/R alone. n = 6.

Furthermore, echocardiography was performed to determine the effects of GRS on cardiac performance. As noticed in Fig. 2, compared to sham, MI/R significantly impaired LVEF and LVFS, while did not markedly affect LV Mass and LV volumes. By contrast, GRS and captopril markedly attenuated MI/R-induced impairment of LVEF and LVFS, and GRS at concentration of 19.2 mg/kg exhibited the similar function compared to YQFM, whereas also showed no significant difference on LV Mass and LV volumes. Collectively, these data suggest that GRS treatment reduces infarct size and improves cardiac performance in the model of MI/R injury.

### GRS inhibited myocardial apoptosis in MI/R mice

**Figure 3:**
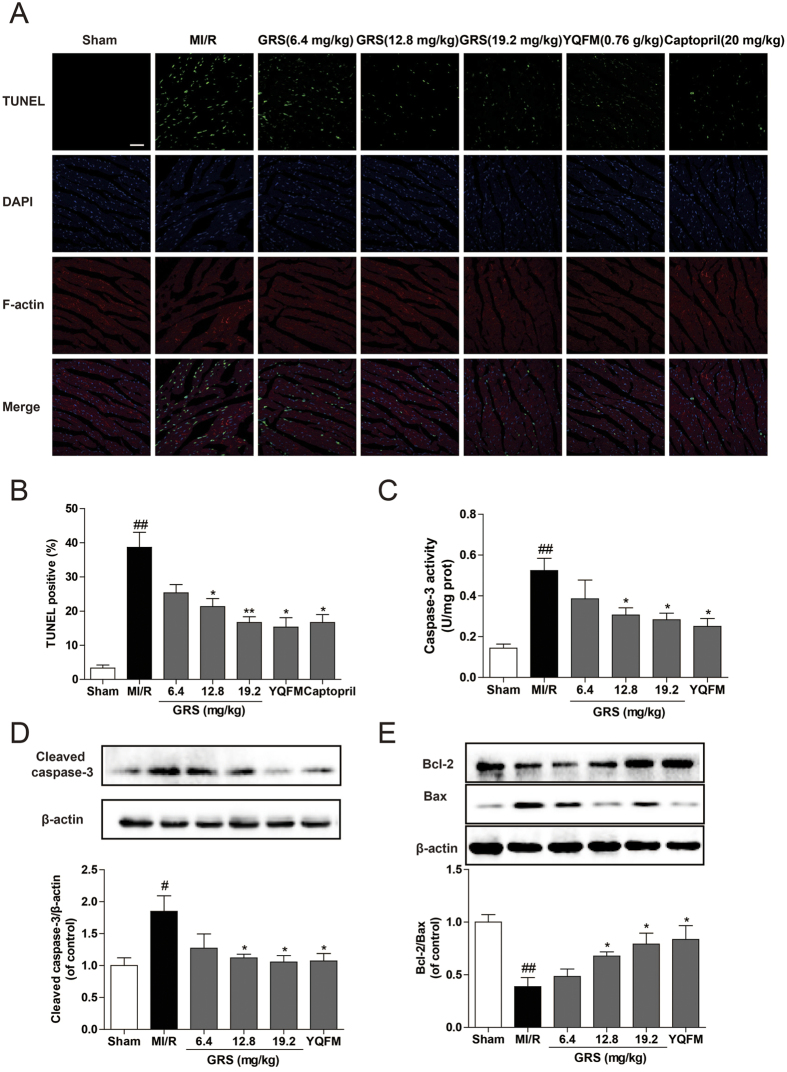
GRS inhibited myocardial apoptosis in MI/R mice. (**A**) Representative photomicrographs of TUNEL staining images (Bar = 40 μm). Total nuclei were labeled with DAPI (blue), F-actin was labeled in red, and apoptotic nuclei were detected by TUNEL staining (green). n = 3. **(B)** Quantitative analysis of apoptotic cells in indicated groups. **(C)** Activity of caspase-3 was measured through the specific cleavage of substrates in each group. n = 6. **(D)** Protein expression levels of cleaved caspase-3 was determined by Western blot analysis. n = 3. **(E)** Protein expression levels of Bcl-2 and Bax were determined by Western blot analysis. n = 3. Results were obtained from three independent experiments and were presented as mean ± SEM. ^#^*P* < 0.05, ^##^*P* < 0.01 vs. Sham group without MI/R, *P < 0.05, **P < 0.01 vs. group treated with MI/R alone.

Myocardial apoptosis is an essential contributor to infarct size expansion and cardiac dysfunction after ischemic reperfusion injury^23^. Therefore, we performed TUNEL staining to explore whether GRS prevented MI/R-induced myocardial apoptosis. Following MI/R, vehicle-treated mice exhibited obvious TUNEL-positive (apoptosis) cells in ischemic myocardium compared to sham treated mice (Fig. 3A,B). By contrast, a significantly lower proportion of apoptotic cells was observed in heart slices from GRS-treated mice. Furthermore, as shown in Fig. 3C–E, MI/R resulted in a noticeable increase in caspase-3 activity and expression, and the decrease of Bcl-2/Bax ratio. While GRS treatment suppressed caspase-3 activity and expression significantly, meanwhile, up-regulated the expression of Bcl-2/Bax ratio compared with that of MI/R group. These data suggest a potent anti-apoptotic effect of GRS in MI/R injury, and exhibit the similar effect compared to YQFM.

### GRS attenuated H/R-induced injury and apoptosis in cardiomyocytes

We first examined the viability of cardiomyocytes after treatment with GRS and we found that GRS at concentrations of 0.1–60 μg/mL did not significantly affect cardiomyocytes viability (Supplementary Fig. S1A,B). On the basis of the results of the previous step, three concentrations (0.1, 1 and 10 μg/mL) were chosen for further investigation. Exposure of H9c2 cardiomyocytes (H 6 h/R 6 h) and primary cardiomyocytes (H 12 h/R12h) to H/R led to a decrease in cell viability, whereas treatment with 0.1–10 μg/mL GRS maintained cell viability (Supplementary Fig. S1C,D). As LDH release was an accepted marker for cell damage, cardiomyocytes injury was also investigated by determining the release of LDH in culture medium. As compared to control group, LDH release markedly increased after H/R injury, while treatment with 0.1–10 μg/mL GRS significantly inhibited the release of LDH (Supplementary Fig. S1E,F). Clearly, these data indicated that GRS with the highest concentration exhibited the same efficacy as YQFM.

**Figure 4:**
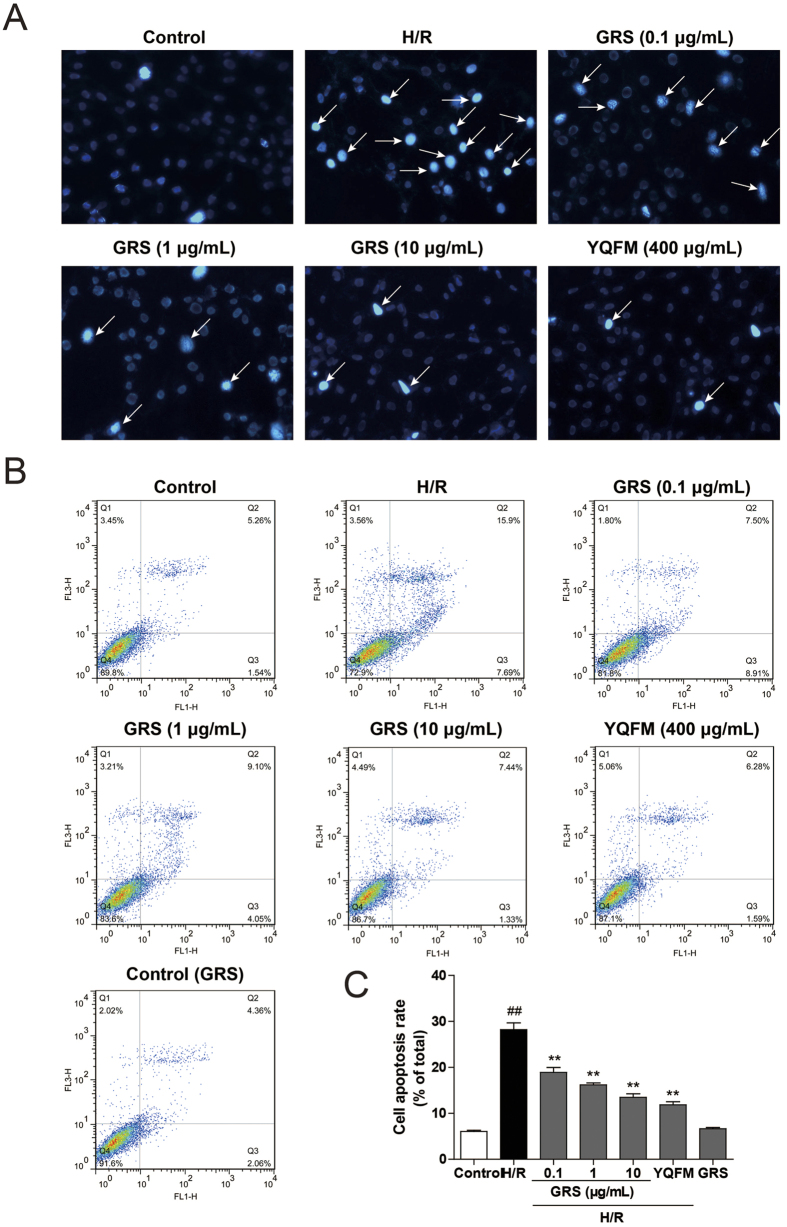
GRS attenuated H/R-induced H9c2 cardiomyocytes apoptosis. H9c2 cells were treated with GRS at the concentration of 0.1–10 μg/mL and then exposed to hypoxia of 6 h followed by 6 h reoxygenation. **(A)** Cell apoptosis was detected by Hoechst 33342 staining (400×magnification). The arrows indicate the representative apoptotic nuclei. **(B)** H/R-induced cell apoptosis rate was quantified by Flow Cytometry. **(C)** Quantitative analysis of apoptotic cells in indicated groups. Results were obtained from three independent experiments and were presented as mean ± SEM. ^##^*P* < 0.01 vs. control group without H/R, **P < 0.01 vs. group treated with H/R alone.

Nuclear morphological change was investigated by Hoechst 33342 staining, which observed that the control group appeared uniformly dispersed chromatin, normal organelles and intact cell membranes. Following H/R, the nuclei exhibited irregularly shaped and hyper-condensed (brightly stained). Whereas the morphological changes were significantly alleviated with GRS treatment, and the number of cells with nuclear condensation and fragmentation was significantly decreased (Fig. 4A). Quantitative analysis using flow cytometric further confirmed that the apoptotic index was markedly increased compared with that of control group. However, the apoptotic index was significantly decreased by treatment with GRS (Fig. 4B,C). The anti-apoptotic effect of GRS with the concentration at 10 μg/mL was the same as YQFM (400 μg/mL).

### GRS restored H/R-induced loss of mitochondrial membrane potential and reduced cardiomyocytes apoptosis

**Figure 5:**
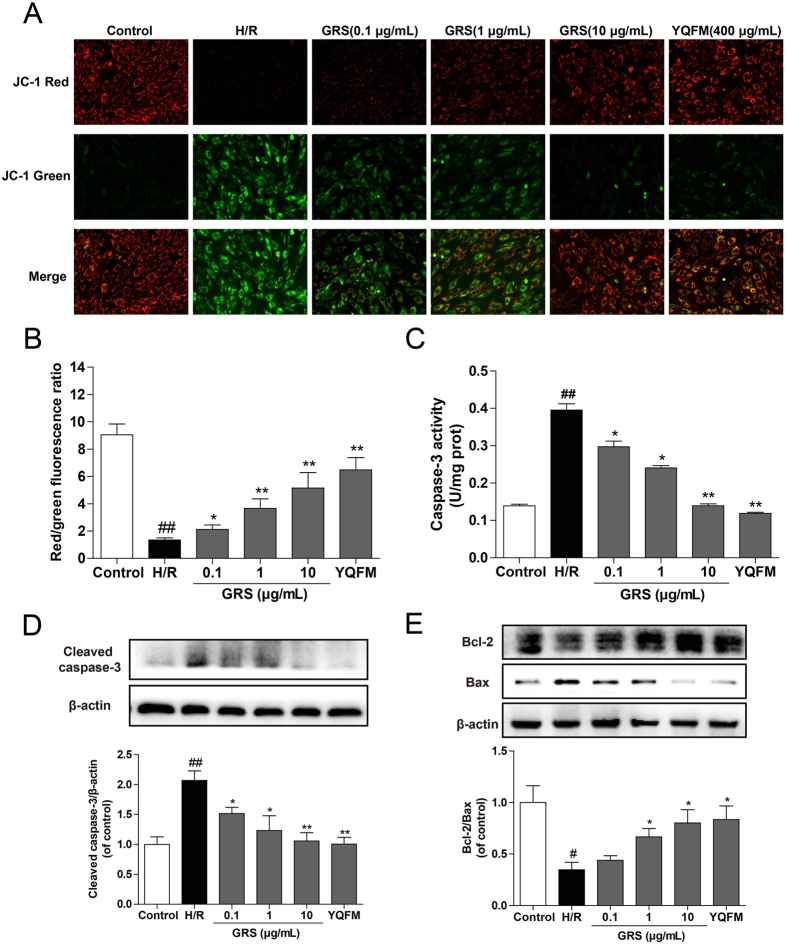
GRS restored H/R-induced loss of mitochondrial membrane potential and reduced cardiomyocytes apoptosis. H9c2 cardiomyocytes were treated with GRS at the concentration of 0.1–10 μg/mL and then exposed to hypoxia of 6 h followed by 6 h reoxygenation. **(A)** Mitochondrial membrane potential was assessed by the lipophilic cationic probe JC-1 (400×magnification). Red signal indicates JC-1 aggregates in mitochondria. Green signal shows cytosolic JC-1 monomers indicative of the loss of mitochondrial membrane potential. **(B)** Quantitative analysis of membrane potential in (A). **(C)** Activity of caspase-3 was measured through the specific cleavage of substrates in each group. **(D)** Caspase-3 protein expression was detected by western blot. **(E)** Bcl-2 and Bax expression were detected by western blot. Results were obtained from three independent experiments and were presented as mean ± SEM. ^#^*P* < 0.05, ^##^*P* < 0.01 vs. control group without H/R, *P < 0.05, **P < 0.01 vs. group treated with H/R alone.

As shown in Fig. 5A,B, control group exhibited bright-staining mitochondria that emitted red fluorescence. Cells exposed to H/R caused the formation of monomeric JC-1, indicative of loss of membrane potential. While GRS treatment blocked the H/R-induced formation of JC-1 monomers, suggesting GRS could restore H/R-induced loss of mitochondrial membrane potential. Moreover, caspase-3 was an essential downstream enzyme of the final step of apoptotic pathway, and it could cause DNA degradation and apoptosis when it was activated. As illustrated in Fig. 5C,D, H/R led to a noticeable increase in caspase-3 expression and activity compared with that of control group, while treatment GRS reduced the level of caspase-3 significantly. The balance of anti- and pro-apoptotic proteins in Bcl-2 family plays a significant role in the control of cell survival against ischemia reperfusion injuries^24^. As presented in Fig. 5E, the Bcl-2/Bax ratio was markedly increased in GRS treated cells compared to the H/R group. We further observed H/R-induced primary cardiomyocytes apoptosis in the presence of GRS. And we found that GRS upregulated Bcl-2 expression and downregulated Bax, active caspase-3 expression and activity in primary cardiomyocytes injured by H/R (Supplementary Fig. S2). These results supported the anti-apoptotic effect elicited by GRS with the similar efficacy as YQFM.

### GRS regulated Drp1 phosphorylation, translocation and prevented mitochondrial fission

**Figure 6:**
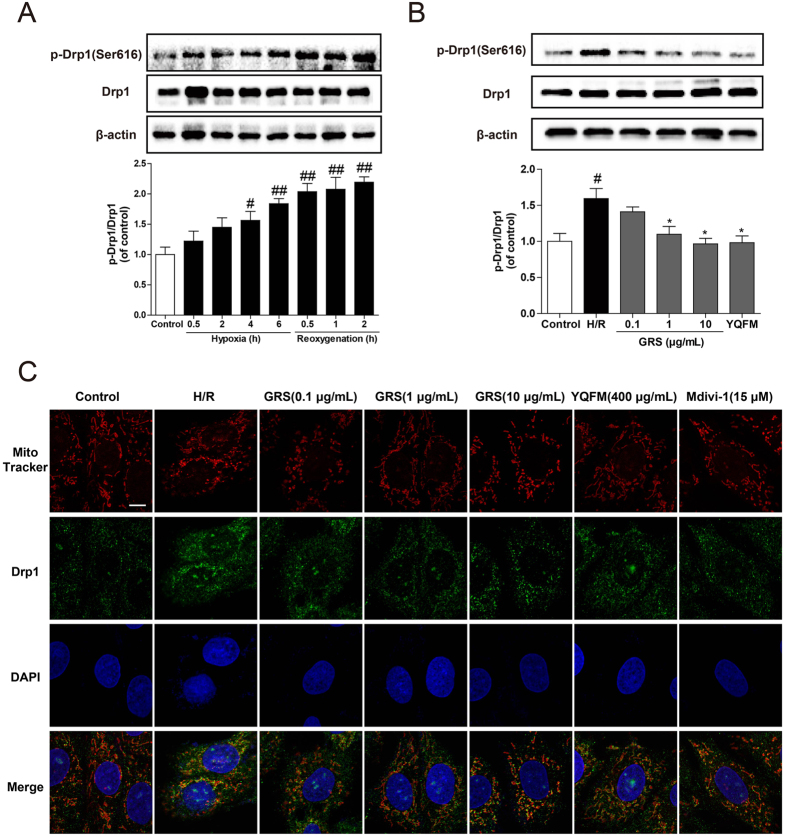
GRS regulated Drp1 phosphorylation, translocation and prevented mitochondrial fission. **(A)** Effects of hypoxia and reoxygenation on expression of Drp1 and p-Drp1 (Ser616) in H9c2 cardiomyocytes. The proteins levels were determined using western blot analysis. **(B)** H9c2 cardiomyocytes were treated with GRS at the concentration of 0.1–10 μg/mL and then exposed to hypoxia of 6 h followed by 2 h reoxygenation. Drp1 and p-Drp1 (Ser616) protein expression were detected by western blot. **(C)** View of mitochondrial localization of Drp1 with confocal scanning microscope in H9c2 cardiomyocytes (Bar = 10 μm). Mitochondrial morphology and Drp1 translocation were analyzed using a 63× oil immersion lens. Results were obtained from three independent experiments and were presented as mean ± SEM. ^#^*P* < 0.05, ^##^*P* < 0.01 vs. control group without H/R, *P < 0.05 vs. group treated with H/R alone.

Drp1 regulates mitochondrial morphology by promoting mitochondrial fission, and phosphorylation of Drp1 at Ser616 stimulates mitochondrial fission. As shown in Fig. 6A, there was no alteration in total Drp1 protein abundance during H/R injury, while an immunoblot of p-Drp1 at Ser616 showed a remarkable increase for a period of 4 and 6 h after hypoxia, and 0.5, 1, 2 h after reoxygenation. A statistic test revealed that the expression was significantly increased in a time-dependent manner after reoxygenation and we chose the time point of reoxygenation of 2 h to further explore the efficacy of GRS. We found that GRS at concentration of 1–10 μg/mL markedly decreased Drp1 phosphorylation at Ser616 in H9c2 cardiomyocytes (Fig. 6B) and primary cardiomyocytes (Supplementary Fig. S3A) exposed to H/R injury. Also, GRS significantly inhibited myocardial Drp1 phosphorylation at Ser616 in MI/R injury (Supplementary Fig. S4A).

We further investigated the changes in mitochondrial morphology by MitoTracker Red staining. Mitochondria are organized in a highly dynamic tubular network, while cells exhibiting fragmented or spherical mitochondria undergo mitochondrial fission in response to H/R injury. The translocation of Drp1 from cytosol to mitochondria is an indicator for Drp1 activation. GRS inhibited H/R-induced mitochondrial fission and reduced the location of Drp1 at mitochondria in cardiomyocytes (Fig. 6C, Supplementary Fig. S3B), indicating the inhibition of Drp1 recruitment, which showed the same effect of YQFM and the mitochondrial fission inhibitor Mdivi-1.

### GRS inhibited mitochondrial fission with regulation of AMPK

**Figure 7:**
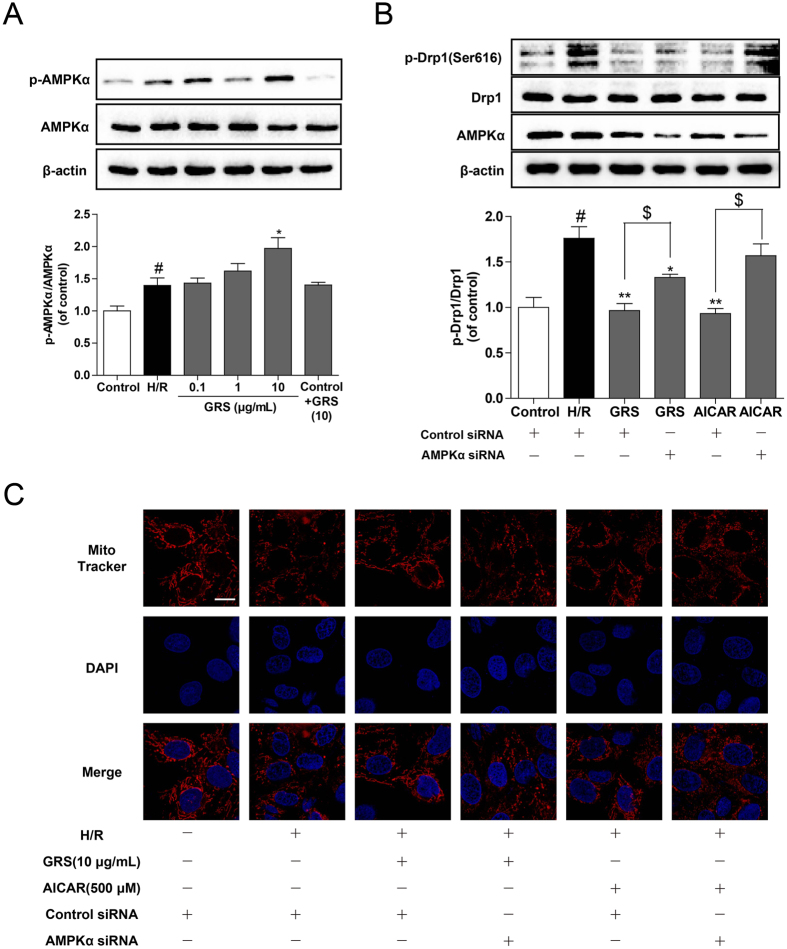
GRS inhibited mitochondrial fission with regulation of AMPK. (**A**) H9c2 cardiomyocytes were treated with GRS at the concentration of 0.1–10 μg/mL and then exposed to hypoxia of 6 h followed by 1 h reoxygenation. AMPKα and p-AMPKα expression were detected by western blot. **(B)** GRS and AICAR incubated with H/R injury in H9c2 cardiomyocytes transfected with AMPKα or control scrambled siRNAs. AMPKα, Drp1 and p-Drp1 expression were detected by western blot. **(C)** Mitochondrial fission was detected by Mito Tracker Red with confocal microscopy (Bar = 10 μm). ^#^*P* < 0.05 vs. control group without H/R, *P < 0.05, **P < 0.01 vs. group treated with H/R alone, ^$^*P* < 0.05 vs. group treated with H/R and AMPKα siRNA.

As AMP-activated protein kinase (AMPK) agonist is observed to modulate Drp1 phosphorylation in the endothelium^11^, we wondered whether this regulation was involved in the action of GRS and whether activation of AMPK regulated mitochondrial fission in cardiomyocytes exposed to H/R injury. First, we observed the effect of GRS on AMPK activity in cardiomyocytes. GRS treatment significantly increased AMPKα phosphorylation in cardiomyocytes and MI/R injured mice (Fig. 7A, Supplementary Figs S4B and S5A). As choosing H9c2 cell line for siRNA transfection is more feasible than using primary cardiomyocytes, we treated H9c2 cells with specific AMPKα siRNA. The siRNA transfection was optimized and total cell lysates were subjected to SDS-PAGE for immunoblotting analysis with β-actin as a reference to evaluate the efficiency of AMPKα siRNA. And AMPKα was successfully knocked down by a siRNA approach with the inhibition efficacy larger than 60% (Supplementary Fig. S5B–D).

Meanwhile, as shown in Fig. 7B, we found that AMPK agonist AICAR with concentration 500 μM inhibited Drp1 phosphorylation at Ser616 and silencing AMPKα attenuated the positive effect of AICAR on Ser616 phosphorylation, indicating that AMPK activation was essential in the regulation of Drp1 phosphorylation. In addition, we found that silencing of AMPKα partly alleviated the inhibitory action of GRS on Drp1 phosphorylation at Ser616. Moreover, AMPKα knockdown attenuated the positive effect of GRS on H/R-induced mitochondrial fission in cardiomyocytes (Fig. 7C). These results supported our hypothesis that AMPK activation regulated mitochondrial fission and was essential for the inhibitory action of GRS in Drp1 activation and mitochondrial fission in cardiomyocytes.

## Discussion

There have been a number of studies illustrating the clinical usefulness of Shengmai preparations in the treatment of cardiovascular diseases, such as YiQiFuMai powder injection and Shengmai injection. Several main types of chemical components, for instance, ginsenosides, lignans and homoisoflavonoids have been suggested to be responsible for the pharmacological activities of Shengmai preparations^25^. Nowadays, the safety and efficacy of TCM injections have been a focus of attention for the medical community. Therefore, investigations on their bioactive components and mechanisms of action are significant to determine the quality control of TCM injections. It is necessary to develop some safer and more efficient compositions basing on these effective components in TCMs.

In our previous study, we chose three representative components (GRS-ginsenoside Rb1, ruscogenin and schisandrin) for the optimization design investigation, using quantitative pharmacological methods on the basis of preliminary studies and relevant literatures about Shengmai preparations for myocardial ischemia, and this combination had obtained an authorized patent in China^21^. Although reperfusion therapies have markedly reduced ischemia-induced injury and mortality, effective intervention strategies still remain limited^26^. In the present study, treatment with GRS significantly reduced infarct size and preserved cardiac function in mice subjected to MI/R injury. These positive effects of GRS were associated with suppression of mitochondrial mediated apoptosis and mitochondrial fission. Furthermore, GRS upregulated expression of AMPKα, while the protective effects of GRS were significantly blunted by knockdown of AMPKα. Collectively, these data indicate that pharmacological activation of AMPK inhibits mitochondrial fission via dephosphorylation of Drp1 at Ser616 in cardiomyocytes, and GRS is a potent TCM drug combination which reduces MI/R injury by suppressing mitochondrial mediated apoptosis and fission via upregulating AMPKα.

Accumulating evidence suggests that the dropout of cardiomyocytes as a result of apoptosis is vital in various heart diseases and inevitably leads to heart failure^27^. Myocardial apoptosis is a major determinant of MI/R-induced cardiac dysfunction. It is widely acknowledged that the intervention of apoptosis process could inhibit the loss of cardiomyocytes, minimize cardiac injury and eventually slow down or even prevent the occurrence and development of MI/R injury^28^.

Consistent with our previous study^10^, the present study demonstrated the protective effect of Shengmai preparations YQFM on MI/R injury. Similar to YQFM, the active ingredients combination GRS also exerted cardioprotective effect with the same potency, as shown by improved LV function, decreased infarct size, cell viability loss and LDH activities. In addition, the anti-apoptotic effect of GRS was also validated by the results of TUNEL staining, Hoechst 33342 staining and Annexin-V/PI staining. These results strongly suggested that GRS might exert a protective effect against MI/R injury and inhibit cardiomyocytes apoptosis in response to MI/R injury, which laid the foundation for the further development of new drug with independent intellectual property.

I/R injury also generated prominent variations in cell signaling, including disturbance of mitochondrial membrane potential, caspase-3 activation and imbalance of anti- and pro-apoptotic proteins in Bcl-2 family proteins, indicating an activation of mitochondrial apoptosis pathway. Mitochondria not only provides energy for cardiomyocytes but also plays an essential role in cardiomyocytes apoptotic signaling pathway. Mitochondria dysfunction is a predominant cause of I/R induced cardiomyocytes apoptosis, as evidenced by decreased mitochondrial membrane potential as an indicator of MPTP opening, as reported previously^29^,^30^. This disturbance may be due to the altered mitochondria transport of proton in electron transport chain and manifested as de- and hyper-polarization^31^. Nevertheless, GRS treatments restored mitochondrial membrane potential. It is well known that mitochondrial permeability and release of the apoptosome could be controlled by Bcl-2 family^32^. An increase of pro-apoptotic proteins Bax and a decrease in anti-apoptotic proteins Bcl-2 could lead to a decrease in mitochondrial membrane potential and an opening of mitochondrial permeability transition pores, leading to cytochrome c release from mitochondria into cytosol^33^. Therefore, the ratio of Bcl-2 to Bax is a critical factor in the cellular threshold for apoptosis. The current research showed that GRS markedly inhibited Bax expression but increased Bcl-2 expression under the H/R condition, eventually resulting in an increased Bcl-2/Bax ratio and cell survival rate. Moreover, caspase cascade also plays an essential role in apoptosis^34^. Our results indicated that GRS resulted in active caspase-3 expression and activity downregulated. Based on the above results, it could be reasonably speculated that GRS might attenuate cardiomyocytes apoptosis, with the similar efficacy as Shengmai preparations of YQFM.

On the basis of the obtained results that GRS protected cardiomyocytes apoptosis induced by I/R, further exploration was conducted with focus on the potential mechanisms involved in the anti-apoptotic effects of GRS. Mitochondrial are dynamic organs constantly undergoing fission and fusion, which are controlled by critical regulatory proteins. Drp1 activation is responsible for H/R-induced mitochondrial dysfunction^35^. The cytoplasmic Drp1 is originally located in the cytosol, and when it is activated via phosphorylation of Drp1 at Ser616, it translocates to the foci of future mitochondrial fission sites, leading to fission and subsequent mitochondrial dysfunction^36^. While Drp1 phosphorylation at the residue of Ser637 is demonstrated to inactivate Drp1^37^. In the present study, H/R injury evoked Drp1 activation, leading to mitochondrial fission. GRS inhibited Drp1 activation by modulation of phosphorylation (Ser616) and thereby protected mitochondrial functional and structural integrity.

AMPK is a serine/threonine kinase, therefore, its direct regulation should be phosphorylation at the serine/threonine residue of target substrate. Recent studies showed that pharmacological activation of AMPK prevents mitochondrial fission by enhancing Drp1 phosphorylation (Ser 637) in vessel endothelial cells^11^. As a specific inhibitor for Drp1-mediated mitochondrial fission, Mdivi-1 protected the heart against ischemia/reperfusion injury^35^, suggesting the possible role of Drp1 as the more possible direct target of AMPK. As expected, our findings illustrated that AMPK activation inhibited Drp1 phosphorylation at Ser616 and mitochondrial fission in H/R-induced cardiomyocytes injury. Meanwhile, knockdown of AMPKα significantly increased phosphorylation of Drp1 at Ser616, suggesting the critical role of AMPKα in H/R-induced cardiomyocytes mitochondrial fission. GRS exerted the ability to activate AMPKα and AMPKα siRNA knockdown attenuated its action in the regulation of Drp1 phosphorylation, indicating that AMPKα activation was essential for its function in the prevention of mitochondrial fission. Moreover, AMPK could be activated through multiple factors, such as the upstream kinases CaMKK, LKB1, PKA, or the ratio of (AMP+ADP)/ATP, and also might be activated through some other proteins which have not been reported so far. Further investigations will be focus on the reason how the GRS activates AMPK.

In conclusion, we have demonstrated that GRS protected myocardium from MI/R injury and inhibited MI/R induced cardiomyocytes apoptosis with the equivalence as ShengMai preparations. More importantly, we found that activation of AMPK improves mitochondrial morphology and function by regulation of Drp1 phosphorylation (Ser616) and recruitment under cardiomyocytes H/R injury. And these results indicated that GRS exerted cardioprotective effect, at least in part, through activating AMPK phosphorylation and improving mitochondrial function in I/R-induced cardiac injury. These findings might provide some pharmacological evidences for further development of new modern Chinese drug for cardiovascular diseases basing on traditional Chinese formula with affirmative therapeutic effect.

## Materials and Methods

### Drugs and reagents

GRS was a mixture of ginsenoside Rb1, ruscogenin and schisandrin (6:0.75:6). Ginsenoside Rb1 and schisandrin were purchased from Nanjing Zelang Bio-Technology Co., Ltd (Nanjing, China). Ruscogenin was isolated in our laboratory and the purity was determined to be higher than 99% by HPLC. 2,3,5-triphenyltetrazolium chloride (TTC), Mdivi-1, DNase, Cytosine β-D-arabinofuranoside (AraC), Poly-D-Lysin (PDL) and AICA riboside (AICAR) were purchased from Sigma-Aldrich (St. Louis, MO, USA). The kits for determination of lactate dehydrogenase (LDH), caspase-3 activity and fluorescent kit for Hoechst 33342, probe JC-1 were obtained from Beyotime Institute of Biotechnology (Shanghai, China). FITC Annexin V apoptosis detection kit was from Becton Dickinson Co. (San Diego, CA, USA). Mito-Tracker^®^ Deep Red FM was purchased from life technologies (California, USA). Dulbecco’s modified Eagle medium (DMEM) was obtained from GIBCO/BRL (Life Technologies, California, USA). Fetal bovine serum (FBS) was from ScienCell (Carlsbad, CA, USA). RIPA lysis buffer, protease inhibitor and enhanced chemiluminescene (ECL) reagent were from Vazyme Biotech (Nanjing, China). AMPKα siRNA kit was from Santa Cruz Biotechnology (Santa Cruz, CA, USA). Antibody against β-actin was from Bioworld Technology (St. Louis Park, MN, USA), antibody against caspase-3, Bcl-2, Bax, Drp1, phospho-Drp1 (Ser616), AMPKα and phospho-AMPKα (Thr-172) were obtained from Cell Signaling Technology (Boston, MA, USA).

### Animals and MI/R injury model

Male ICR mice (22–25 g) were purchased from Model Animal Research Centre of Yangzhou University (Yangzhou, Jiangsu, China). The animals were housed in a standard vivarium with free access to food and water. Prior to experiments, animals were randomized into experimental groups. All procedures were approved by the Animal Ethics Committee of China Pharmaceutical University, and the Laboratory Animal Management Committee of Jiangsu Province (Approval No.: 2121961). All methods for *in vivo* study were carried out in accordance with the approved guidelines. The MI/R model was generated as previously described^38^. Briefly, mice were anesthetized intraperitoneally with chloral hydrate (400 mg/kg). For myocardial ischemia, a slipknot was tied around the left anterior descending coronary artery 3–4 mm from its origin utilizing a 6–0 silk suture. After 30 min of ischemia, the slipknot was released and followed by 24 h of reperfusion. ST-segment elevation on an electrocardiogram monitor represented a success in MI/R model performance. Sham-operated animals were subjected to the same surgical procedures without ligating left anterior descending coronary artery. All drugs were administered via intraperitoneally within 10 min at the beginning of reperfusion. The surgery was performed by a technician blinded to treatment assignment. No difference was observed in surgical mortality among groups investigated and the survival rate of animals was more than 85%.

### Measurement of myocardial infarct size

Twenty-four hours after reperfusion, the heart (n = 6) was quickly excised, frozen at −70 °C, and the ventricular tissue was cut into five slices perpendicular to the long axis of the heart. The heart sections were then incubated individually using a 24-well culture plate with 1% TTC solution at 37 °C for 15 min, and photographed digitally. Red parts in the heart stained by TTC indicated ischemic but viable tissue. While staining negative areas represented infarcted myocardium. Areas of infarct size were measured by computerized planimetry. The size of infarction area was expressed as percentage of the total LV area.

### Measurement of LDH in serum and histopathologic examination

Myocardial damage was evaluated by measuring plasma concentration of LDH. At the end of the experiment, blood was collected and serum was separated by centrifugation, the LDH was detected as previously described^10^. After collecting the blood samples, the hearts were removed. Heart tissues (n = 6) were fixed in 10% buffered paraformaldehyde solution, embedded in paraffin, sliced into pieces of 5 μm thick, and stained with hematoxylin and eosin (H&E). The histopathological changes were detected by optical microscope.

### Echocardiographic measurement

Cardiac function was assessed noninvasively at 24 h after MI/R using an echocardiographic system consisting of a Vevo 2100 Imaging System (Visual Sonics, Toronto, Canada) equipped with a 30 MHz transducer. The mice (n = 6) were anesthetized using 2.5% isoflurane in O_2_ gas. When fully anesthetized, each mouse was transferred to dorsal recumbency and placed on a heated imaging platform. The following parameters were measured as indicators of cardiac function: Left ventricular (LV) ejection fraction (EF), LV fractional shortening (FS), LV mass, LV volumes, LV internal dimensions at diastole (LVIDD), LV posterior wall dimensions at diastole (LVPWD) and interventricular septal dimensions at diastole (IVSDD).

### TUNEL staining for apoptosis *in vivo*

At 24 h after MI/R, ischemic/reperfused myocardium (n = 3) was harvested and fixed in 4% paraformaldehyde solution. Terminal deoxynucleotidyl transferase-mediated deoxyuridine triphosphate nick-end labeling (TUNEL) staining was carried out to assess myocardial apoptosis using the Fluorescein *In Situ* Cell Death Detection Kit (Roche Diagnostics, Indianapolis, IN) as previously described^28^. Cardiomyocytes, apoptotic nuclei and total cardiomyocyte nuclei were labeled with anti-F-actin antibody, green fluorescein staining and DAPI, respectively. For each slice, 5 fields were randomly obtained under a confocal scanning microscope (LSM700, Zeiss, USA). Extent of cell apoptosis was expressed as ratio of TUNEL positive nuclei over DAPI-stained nuclei.

### Determination of caspase-3 activities *in vivo*

Caspase-3 activity was measured using a colorimetric activity assay kit as previously described^39^. Heart tissues (n = 6) were obtained from the margin of infarct areas, lysed in ice-cold lysis buffer for 30 min, then centrifuged at 4 °C for 10 min at 12,000 rpm. The supernatant was incubated with the caspase-3 substrate (Ac-DEVD-*p*NA) after quantification of protein concentration. The caspase-3 activity was determined using a Microplate Reader at 405 nm.

### Cell preparation and culture

Neonatal rat ventricular myocytes (NRVMs) were prepared as previously described^40^. Briefly, ventricular myocytes were enzymatically dissociated and enriched for cardiomyocytes. Using this method, we routinely obtained contractile myocardial cell cultures with 98–99% myocytes, as evidences by microscopic observation of cell beating, and by immunofluorescence with cardiac troponin T. Cells were cultured in DMEM supplemented with 10% FBS at 37 °C in a humidified atmosphere of 5% CO_2_.

Rat H9c2 cardiomyocyte cell line was obtained from Shanghai Institute of Cell Biology, Chinese Academy of Sciences (Shanghai, China). The H9c2 cells were maintained in DMEM supplemented with 10% FBS, 100 U/mL penicillin and 100 μg/mL streptomycin at 37 °C in a humidified atmosphere of 5% CO_2_. The culture medium was replaced every 2 days, and cells were subcultured or subjected to experimental procedures at 80–90% confluence.

### H/R injury model *in vitro*

To mimic the ischemic injury *in vitro*, the oxygen and glucose deprived (OGD) technique was established based on a previously described protocol^41^. The OGD injury was produced by incubating with none glucose DMEM and exposed to a hypoxic environment of 94% N_2_, 5% CO_2_ and 1% O_2_ at 37 °C in a humidified N_2_/CO_2_ incubator and then in a standard incubator with 5% CO_2_ in normal atmosphere at 37 °C. All drugs were given at the beginning of hypoxia and retained in the culture medium over the period of H/R.

### Cell viability and LDH assays

Cell viability was determined using MTT assay. After different treatments, cells were incubated with MTT at a final concentration of 0.5 mg/mL for 3 h at 37 °C. Then, the medium was discarded and 150 μL DMSO was added to dissolve the formazan crystals. The absorbance was read at 570 nm with a reference wavelength of 650 nm using a microplate reader and cell viability was expressed as percentage of absorbance to control values. To further measure the extent of cell injury, release of LDH was also detected. At the end of the incubation period, the culture supernatants were collected and the activity of LDH was tested at 490 nm as previously described^42^.

### Hoechst 33342 staining assay

Briefly, H9c2 cardiomyocytes were washed with ice-cold PBS and exposed to Hoechst 33342 (5 μg/mL) and incubated for 30 min at 37 °C. Then the H9c2 cardiomyocytes were washed three times with PBS and observed under a fluorescence microscope (Zeiss Germany). The nuclei of apoptotic cells exhibited irregularly shaped and hyper-condensed (brightly stained).

### Flow-cytometric analysis for apoptosis

Fluorescein isothiocyanate (FITC)-conjugated Annexin V and propidium iodide (PI) were utilized to detect apoptotic cells. Double staining with FITC-Annexin V and PI was carried out as previously described^43^. In brief, H9c2 cardiomyocytes were harvested, washed with PBS, resuspended with binding buffer, then FITC-Annexin-V and PI were added in a final concentration of 100 ng/mL. The mixture was incubated for 15 min in the dark at room temperature. Cellular fluorescence was analyzed with a flow cytometer (Becton Dickinson, USA). Data were analyzed using FlowJo software.

### Determination of caspase-3 activities *in vitro*

Caspase-3 activity was determined using a colorimetric activity assay kit as previously described^39^. Briefly, cells were lysed in ice-cold lysis buffer, placed on ice for 30 min, then centrifuged at 4 °C for 15 min at 12,000 rpm. After determining the protein concentration, the supernatant was incubated with the caspase-3 substrate (Ac-DEVD-*p*NA). The caspase-3 activity was determined using a Microplate Reader at 405 nm.

### Measurement of mitochondrial membrane potential

Mitochondrial membrane potential was detected using fluorescent dye JC-1 as previously described^44^. In brief, the cells were incubated with JC-1 staining solution (10 μg/mL) for 20 min at 37 °C after each treatment. The cells were then washed twice with PBS and immediately analyzed using a fluorescence microscope. Data were given as the relative ratio of red to green fluorescence intensity, indicating the level of depolarization of the mitochondrial membrane potential.

### Mitochondrial fission analysis

For mitochondrial fission assay, H9c2 cardiomyocytes and primary cultured cardiomyocytes were treated with GRS, YQFM and Mdivi-1 at given concentrations with or without the H/R injury. After incubation, the cells were washed with PBS, and then incubated with 400 nmol/L Mito Tracker® Deep Red FM (Molecular Probes) for 30 min at 37 °C. The cells were then fixed, permeabilised and incubated with anti-Drp1 primary antibody, followed by incubation with an Alexa Fluor 488 conjugated Donkey Anti-Goat IgG (H+L) antibody and DAPI. The structures of mitochondria were viewed by confocal microscopy.

### siRNA transfection

H9c2 cardiomyocytes were transfected with siRNA directed against AMPK subunits or scrambled siRNA as a control. In brief, the cells were transfected with 80 nmol/L of total siRNA by using Transfection Reagent as previously described^10^. To evaluated the efficiency of the AMPK siRNA, total cell lysates were subjected to SDS-PAGE for immunoblotting analysis with β-actin as a reference.

### Western blot analysis

As reported^45^, cells were lysed in ice-cold RIPA buffer, supplemented with 1 mM PMSF. For assay in heart tissues obtained from the margin of infarct areas, the tissues were homogenized in RIPA buffer. Proteins were obtained by centrifugation at 12, 000 rpm for 10 min at 4 °C and the concentrations were analyzed by the BCA method. Equal amount of proteins (35 μg) were loaded onto a 12.5% SDS-PAGE and transferred to PVDF membranes (Millipore Corporation, Billerica, MA, USA) by electroblotting. The membranes were blocked with 3% BSA in TBS/T and stained with primary antibodies against caspase-3, Bax, Bcl-2, Drp1, p-Drp1 (Ser 616), AMPK, p-AMPK (Thr 172), β-actin (dilution 1:1000, 1:1000, 1:1000, 1:1000, 1:600, 1:1000, 1:1000, 1:2000, respectively; Cell Signaling Technology, Boston, MA, USA) overnight at 4 °C. Membranes were then probed with peroxidase conjugated secondary antibody at a 1: 8000 dilution (Bioworld Technology, Louis Park, MN, USA). The antigen-antibody complexes were then detected with ECL reagent (Vazyme Biotech, Nanjing, China) and visualized by ChemiDoc™ MP System (Bio-Rad) and analyzed using Image Lab™ Software (version 4.1, Bio-Rad).

### Statistical analysis

All experiments were performed in triplicate and data were expressed as the mean ± SEM. Statistical analysis was carried out using Student’s two-tailed t-test for comparison between two groups and one-way analysis of variance (ANOVA), followed by Dunnett’s test when the data involved three or more groups. *P* < 0.05 was defined as significant.

## Additional Information

**How to cite this article**: Li, F. *et al*. Cardioprotection by combination of three compounds from ShengMai preparations in mice with myocardial ischemia/reperfusion injury through AMPK activation-mediated mitochondrial fission. *Sci. Rep.* **6**, 37114; doi: 10.1038/srep37114 (2016).

**Publisher’s note**: Springer Nature remains neutral with regard to jurisdictional claims in published maps and institutional affiliations.

## Supplementary Material

Supplementary Figures
