# Supplementary material for: Cardioprotection by combination of three compounds from ShengMai preparations in mice with myocardial ischemia/reperfusion injury through AMPK activation-mediated mitochondrial fission
Source: Sci Rep. 2016 Nov 21;6:37114. doi: 10.1038/srep37114 (PMC5116669; doi:10.1038/srep37114)
Supplement: Supplementary Figures [file srep37114-s1.pdf]

Cardioprotection by combination of three compounds from ShengMai preparations in mice with myocardial ischemia/reperfusion injury through AMPK activation-mediated mitochondrial fission

Fang Li, Xiaoxue Fan, Yu Zhang, Lizhi Pang, Xiaonan Ma, Meijia Song, Junping Kou*, Boyang Yu**

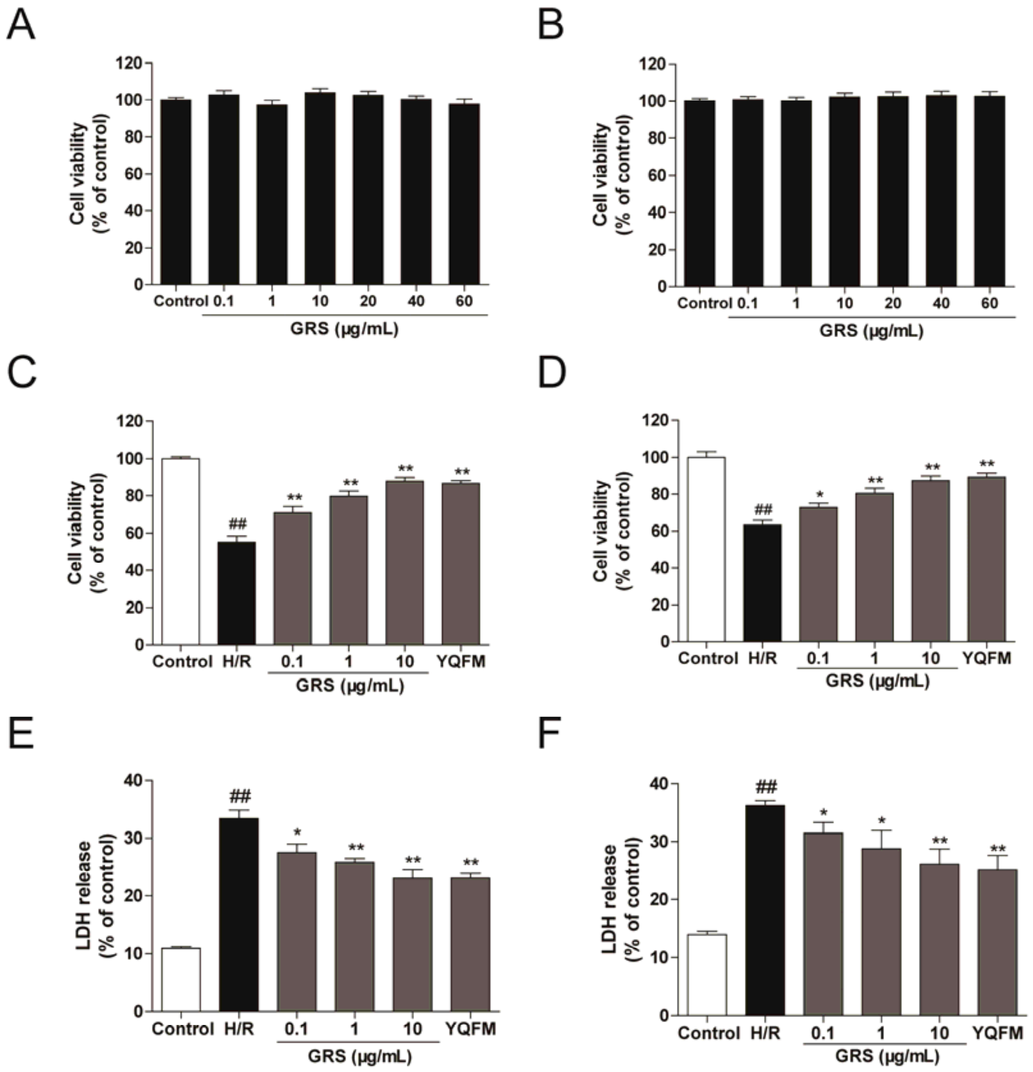

Figure S1. GRS protected cardiomyocytes against injury induced by H/R. H9c2 cells were treated with GRS at the concentration of 0.1-10 μg/mL and then exposed to hypoxia of 6 h followed by 6 h reoxygenation. Primary cardiomyocytes were treated with GRS at the concentration of 0.1-10 μg/mL and then exposed to hypoxia of 12 h followed by 12 h reoxygenation. **(A-B)** Effect of GRS on H9c2 cardiomyocytes (A) and primary cardiomyocytes (B)

viability. **(C-D)** H9c2 cardiomyocytes (C) and primary cardiomyocytes (D) were treated with GRS followed by H/R and cell viability was determined by MTT assay. **(E-F)** The release of LDH in H9c2 cadiomyocytes (E) and primary cardiomyocytes (F) culture medium at the end of reperfusion were determined. Results were obtained from three independent experiments and were presented as mean ± SEM. $^{##}P < 0.01$ vs. control group without H/R, $^{*}P < 0.05$, $^{**}P < 0.01$ vs. group treated with H/R alone.

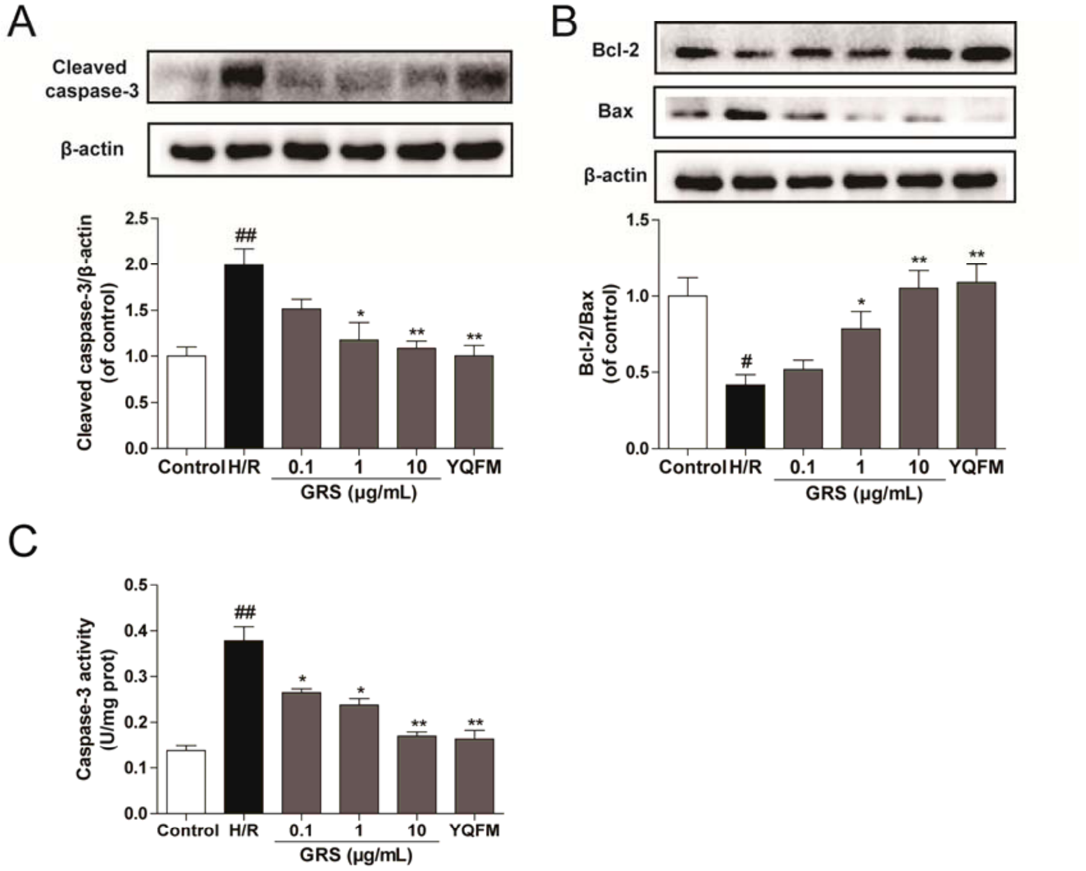

Figure S2. GRS regulated the expression of apoptosis-related proteins and inhibited the caspase-3 activity. Primary cardiomyocytes were treated with GRS at the concentration of 0.1-10 μg/mL and then exposed to hypoxia of 12 h followed by 12 h reoxygenation. **(A)** Caspase-3 protein expression was detected by western blot. **(B)** Bcl-2 and Bax expression were detected by western blot. **(C)** GRS decreased caspase-3 activity in primary cardiomyocytes subjected to H/R. Results were obtained from three independent experiments and were presented as mean ± SEM. $^{#}P < 0.05$, $^{##}P < 0.01$ vs. control group without H/R, $^{*}P < 0.05$, $^{**}P < 0.01$ vs. group treated with

H/R alone.

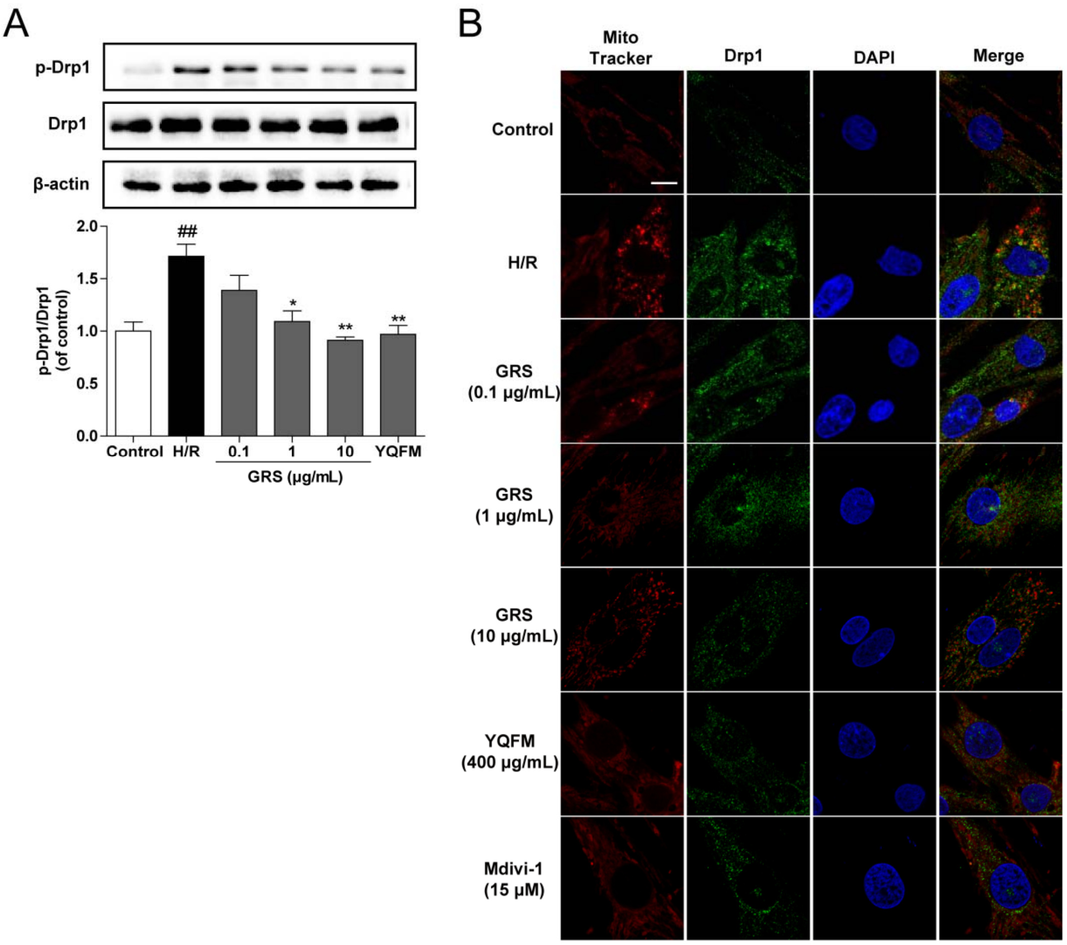

Figure S3. GRS regulated Drp1 phosphorylation, translocation and prevented mitochondrial fission in primary cardiomyocytes. (A) Primary cardiomyoctyes were treated with GRS at the concentration of 0.1-10 μg/mL and then exposed to hypoxia of 12 h followed by 2 h reoxygenation. Drp1 and p-Drp1 (Ser616) protein expression were detected by western blot. **(B)** View of mitochondrial localization of Drp1 with confocal scanning microscope in primary cardiomyocytes (Bar = 10 μm). Results were obtained from three independent experiments and were presented as mean ± SEM. $^{\#\#}P < 0.01$ vs. control group without H/R, $^{*}P < 0.05$, $^{**}P < 0.01$ vs. group treated with H/R alone.

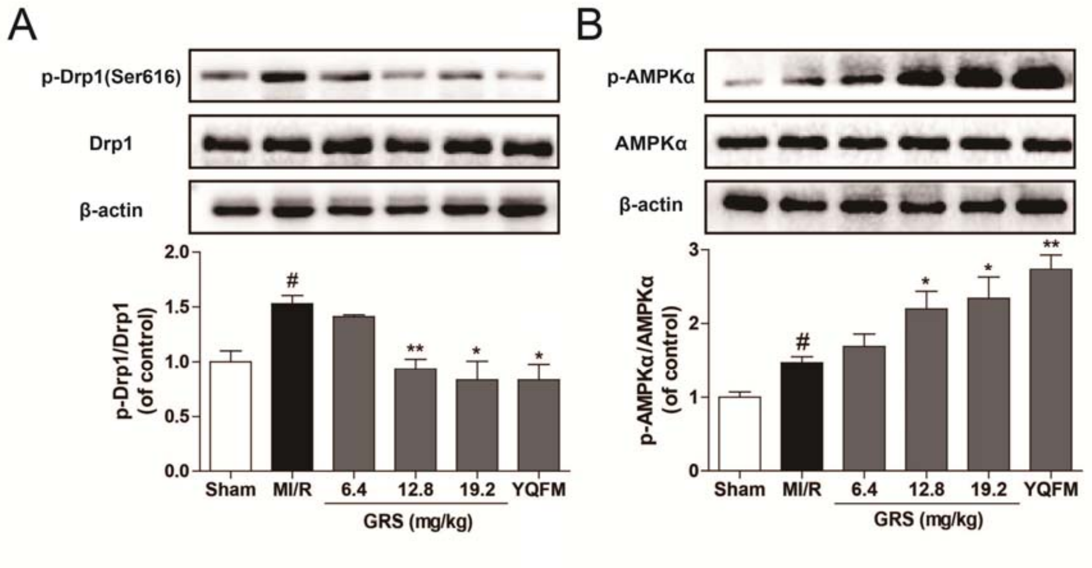

Figure S4. GRS inhibited Drp1 phosphorylation (A) and increased AMPKα phosphorylation (B) in MI/R injured mice. Drp1 and p-Drp1, AMPKα and p-AMPKα protein expression were detected by western blot. Results were obtained from three independent experiments and were presented as mean ± SEM. $^{\#}P < 0.05$ vs. control group without MI/R, $^{*}P < 0.05$, $^{**}P < 0.01$ vs. group treated with MI/R alone. n=3.

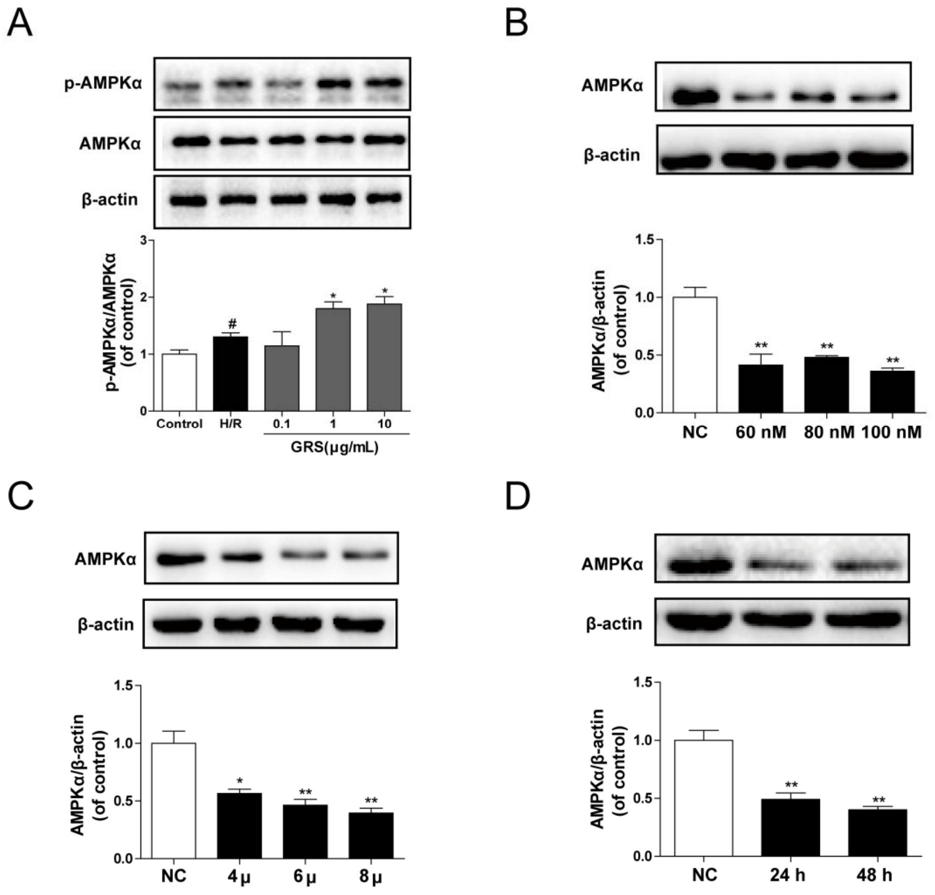

Figure S5. GRS increased AMPKα phosphorylation and the optimization studies of AMPKα siRNA transfection. (A) Primary cardiomyoctyes were treated with GRS at the concentration of 0.1-10 μg/mL and then exposed to hypoxia of 6 h followed by 2 h reoxygenation. AMPKα and p-AMPKα protein expression were detected by western blot. Results were obtained from three independent experiments and were presented as mean ± SEM. $^{\#}P < 0.05$ vs. control group without H/R, $^{*}P < 0.05$ vs. group treated with H/R alone. **(B-D)** The optimizing studies of AMPKα siRNA transfection in the dose of siRNA (B), transfection reagent (C) and the time of transfection (D). Results were obtained from three independent experiments and were presented as mean ± SEM. $^{*}P < 0.05$, $^{**}P < 0.01$ vs. normal control (NC) group.
